# Social Consequences of the COVID-19 Pandemic. A Systematic Review*

**DOI:** 10.17533/udea.iee.v40n1e10

**Published:** 2022-03-30

**Authors:** Pouya Hosseinzadeh, Mordali Zareipour, Esfandyar Baljani, Monireh Rezaee Moradali

**Affiliations:** 1 Student Research Committee. Email: m1997hg@gmail.com Urmia Branch, Islamic Azad University, Urmia, Iran Islamic Azad University Islamic Azad University Urmia Iran m1997hg@gmail.com; 2 Assistant Professor. Khoy University of Medical Sciences, Khoy, Iran. Email: z.morad@yahoo.com. Islamic Azad University Khoy University of Medical Sciences Khoy Iran z.morad@yahoo.com; 3 Ph.D. Assistant Professor. Email: baljanies@gmail.com Urmia Branch, Islamic Azad University, Urmia, Iran Islamic Azad University Islamic Azad University Urmia Iran baljanies@gmail.com; 4 Ph.D. Professor. Email: monir.rezaee@yahoo.co.uk. Urmia Branch, Islamic Azad University, Urmia, Iran Islamic Azad University Islamic Azad University Urmia Iran monir.rezaee@yahoo.co.uk

**Keywords:** COVID-19, coronavirus, pandemics, impacts on health, systematic review., COVID-19, coronavirus, pandemias, impactos en la salud, revisión sistemática., COVID-19, coronavirus, pandemias, impactos na saúde, revisão sistemática

## Abstract

**Objective.:**

To provide a systematic review of the social consequences of COVID-19 pandemic.

**Methods.:**

In the present study, articles indexed in Persian and Latin databases (Web Of Science, Scopus, PubMed, Embase, Google Scholar and Magiran). 43 documents published in the last 3 years in Persian or English language were reviewed. The research steps were performed according to PRISMA writing standard and the quality assessment was done by two researchers independently with Newcastle Ottawa Scale tools for observational studies according to the inclusion criteria.

**Results.:**

Measures to break the chain of virus transmission and to control the COVID-19 pandemic have caused major problems in the economic, social, political and psychological spheres and have affected billions of people worldwide. The COVID-19 pandemic crisis has caused widespread unrest in society and unprecedented changes in lifestyle, work and social interactions, and increasing social distance has severely affected human relations.

**Conclusion.:**

The COVID-19 pandemic has social consequences in certain groups can exacerbate their unfavorable situation. Special groups in crisis situations should be given more attention, and clear and precise policies and programs should be developed to support them.

## Resumen

**Objetivo.:**

Realizar una revisión sistemática de las consecuencias sociales de la pandemia de COVID-19.

**Métodos.:**

En el presente estudio se revisaron los artículos indexados en bases de datos latinas y persas (Web Of Science, Scopus, PubMed, Embase, Google Scholar y Magiran), luego de lo cual se recuperaron 43 documentos publicados en los últimos 3 años en persa o en inglés. Los pasos de la revisión se realizaron según el protocolo PRISMA y, para reducir el sesgo en la evaluación de la calidad, los artículos seleccionados se evaluaron cualitativamente por dos investigadores de forma independiente con la herramienta de Newcastle Ottawa Scale.

**Resultados.:**

Las medidas para romper la cadena de transmisión del virus y para el control de la pandemia de COVID-19 han causado importantes problemas en las esferas económica, social, política y psicológica y han afectado a miles de millones de personas en el mundo. La crisis por la pandemia ha provocado un malestar generalizado en la sociedad y cambios sin precedentes en el estilo de vida, el trabajo y las interacciones sociales. El aumento de la distancia social ha afectado gravemente las relaciones humanas.

**Conclusión.:**

La pandemia de COVID-19 tiene consecuencias sociales que pueden agravar la situación desfavorable de grupos vulnerables. Se debe brindar más atención a los colectivos especiales en situaciones de crisis y desarrollar políticas y programas claros y precisos para apoyarlos.

## Resumo

**Objetivo.:**

Realizar uma revisão sistemática das consequências sociais da pandemia de COVID-19.

**Métodos.:**

No presente estudo, os artigos indexados nas bases de dados latinas e persas (Web Of Science, Scopus, PubMed, Embase, Google Scholar e Magiran) foram revisadas, logo se recuperaram 43 documentos publicados nos últimos 3 anos em persa ou em inglês. Os passos da revisão se realizaram segundo o protocolo PRISMA e, para reduzir o viés na avaliação da qualidade, os artigos selecionados foram avaliados qualitativamente por dois investigadores de forma independente com a ferramenta de Newcastle Ottawa Scale.

**Resultados.:**

As medidas para romper a cadeia de transmissão do vírus e para o controle da pandemia de COVID-19 há causado importantes problemas nas esferas económica, social, política e psicológica e há afetado a milhares de pessoas no mundo. A crise pela pandemia de COVID-19 há provocado um mal-estar generalizado na sociedade e câmbios sem precedentes no estilo de vida, no trabalho e nas interações sociais. O aumento da distância social há afetado gravemente às relações humanas.

**Conclusão.:**

A pandemia de COVID-19 tem consequências sociais que podem agravar a situação desfavorável de grupos vulneráveis. Se deve brindar mais atenção aos coletivos especiais nas situações de crise e desenvolver políticas e programas claros e precisos para apoiá-los.

## Introduction

In late December 2019, an outbreak of a new viral disease belonging to the coronavirus family was reported in Wuhan, the capital of Hubei, China.(1) The new COVID-19 belongs to the same group of coronaviruses as acute respiratory syndrome (SARS) and Middle East Respiratory Syndrome (MERS), which has caused two outbreaks in recent years.(2) The new virus is mainly transmitted by respiratory droplets and contact(3) and affects all age groups.(4) On December, 2021, due to the rapid spread of the virus and the increase in infections, followed by alarming death tolls from the disease was declared an epidemic by the World Health Organization.(5) According to the WHO, as of December 6, 2021, 269 559 230 people worldwide have been infected and 530 5337 people have died from the virus. Also in Iran, as of December 6 in 2021, 6 137 821 people have been infected and 130 277 people have died due to this virus.(6^,^^7^)

The COVID-19 pandemics crisis has caused great unrest in society and unprecedented changes in lifestyle, work and social interactions.(8) The implementation of policies such as social distancing and the closure of gathering and interaction centers such as parks, cafes, shrines, schools, universities, etc., has had certain social consequences.(9) Prolonged stay at home, in a society with a patriarchal lifestyle, will increase the pressure for women to do housework. The economic and psychological dimensions of COVID-19 also affect family members. The issue of increasing domestic violence, including child abuse, spousal abuse, elder abuse, and disability abuse following the COVID-19 pandemics crisis, is such that the Secretary-General of the United Nations has also expressed concern.(10) The closure of schools and universities will deprive millions of children, teenagers and young people of social educational activities for a long time, after which it may not be easy to compensate.(11^,^^12^) Travel restrictions by different countries have reduced social relations in external areas and led to the isolation of individuals. Because COVID-19 affects all aspects of human life, it has increased divorce and reduced marriage in many countries.(13) In couples' lives, we have witnessed a decrease in marital relationships due to fear of contracting or transmitting the disease.(14) COVID-19 has also challenged and damaged public transport.(15^,^^16^)

The COVID-19 greatly affects people's lives. Everyone in the world directly or indirectly faces the severe consequences of this disease. Due to severe isolation and cessation of some social affairs, this disease causes problems such as social anxiety, panic due to insecurity, economic recession and severe psychological stress, which requires coordinated efforts to prevent and control them, and people should follow the advice and the suggestions of government officials and the World Health Organization to make the necessary and at the same time contrary to the internal desire in their daily plan.(17) Given that previous studies have evaluated the psychological and social consequences of other respiratory illnesses; few studies have been performed to evaluate the results of the current epidemic of COVID-19. Therefore, this review study will be conducted with the aim of estimating the social consequences of COVID-19 in order to identify them and take the necessary preventive measures to reduce the problems caused by these consequences. The present study sought to answer the following question: What are the social consequences of COVID-19 disease?

## Methods

Search strategy. All stages of this research were performed based on the writing standard of systematic studies, PRISMA meta-analysis. The study population in this study included articles on the social consequences of COVID-19 that were indexed on one of the Internet sites. The Web of Science, Scopus, PubMed, Embase, and Magiran databases were searched as international databases, and the Google Scholar search engine was searched in Persian and English between 2000 and 2020. To find related articles in Persian and English language databases, the words searched in line with the research topic and based on mesh and syntax were the following items that were combined using AND and OR operators. The searching strategy used in Medline was: “(COVID-19[tiab] OR Coronavirus[tiab] OR Coronaviruses[tiab] OR Deltacoronavirus[tiab] OR Deltacoronaviruses[tiab] OR “Munia coronavirus HKU13”[tiab] OR “Coronavirus HKU15”[tiab] OR (Coronavirus[tiab] AND Rabbit[tiab]) OR “Rabbit Coronavirus*” [tiab] OR (Coronaviruses[tiab] AND Rabbit[tiab]) OR “Bulbul coronavirus HKU11”[tiab] OR “Thrush coronavirus HKU12”[tiab] OR (“Coronavirus 229E”[tiab] AND Human[tiab]) OR (“Coronavirus NL63”[tiab] AND Human[tiab]) OR “Middle East Respiratory Syndrome Coronavirus”[tiab] OR “SARS Virus”[tiab]) AND (“Social Behavior”[tiab] OR (Behavior[tiab] AND Social[tiab]) OR (Behaviors[tiab] AND Social[tiab]) OR “Social Behaviors”[tiab] OR “Competitive Behavior”[tiab] OR “Cooperative Behavior”[tiab] OR Self-Control[tiab] OR “Social Adjustment”[tiab] OR “Social Distance”[tiab] OR “Social Isolation”[tiab] OR “Social Skills”[tiab] OR “Social Stigma”[tiab] OR (Psychology[tiab] AND Social[tiab]))”.

Selection of studies. In total, in this study, systematic review with the above keywords was initially studied in 912 articles and reports, protocols by reputable health organizations and considering the entry and exit criteria of articles in the relevant databases. After studying the titles and abstracts of articles by the authors of the article and removing similar and unrelated items, the relevant items were selected as research. Due to the widespread and increasing prevalence of the disease and the change in the statistics related to the prevalence, the statistics presented in this study are until July 23, 2020. Criteria for selecting articles are: (i) Descriptive, analytical, interventional and review articles related to the last 5 years; (ii) Persian and English language articles published in scientific research journals inside and outside the country, the full text of which was available; and, (iii) Articles related to the study of the social consequences of COVID-19.

**Figure 1.**
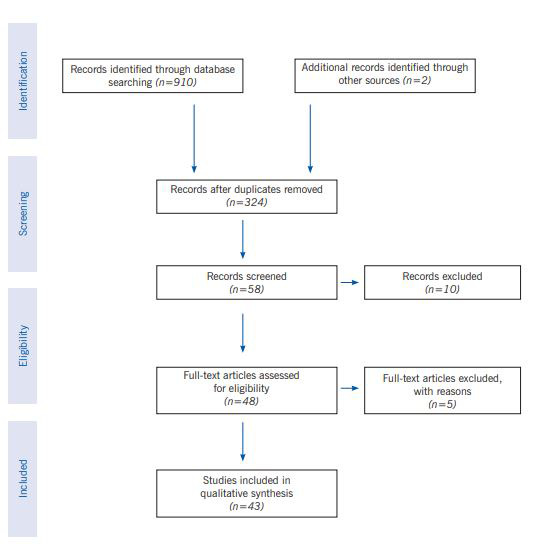
The flowchart describing the study design process

Criteria for deleting articles were: articles that did not have a full text, articles that did not have a clear implementation method, and articles that focused solely on the social consequences of the disease. To review the articles obtained in the search of databases, were evaluated and evaluated according to the inclusion and exit criteria in the working method, four research colleagues participated. After reviewing the inclusion and exclusion criteria of the study, 43 articles and 4 protocols and reports in accordance with the above criteria entered the final quality assessment stage (Figure 1).

Quality control. The quality of the papers selected by the two individuals was assessed independently using the Newcastle Ottawa Scale (NOS). This scale examines articles in terms of selection process (including four sections: sample expediency and sample size, non-response and measurement tools, comparability (control of confounders and influencing factors) and results (evaluation of results and statistical tests). Based on this scale, articles are rated from zero (weakest study) to 10 (strongest study). In this study, studies that scored above 4 were considered as quality studies. Therefore, according to the quality results of the reviewed articles, all articles selected at this stage had a score higher than 4 (optimal level).

Extracting the data: After evaluating the quality, to extract data from the text of articles eligible to enter to the study of two researchers independently by the researcher form, information about each article by name of authors, year of publication, type of research, place of research, Sample size and specificity of samples, standardization of research tools, main findings were extracted. In case of need for review and doubt in the method of reviewing articles, the help of a third researcher was sought (Table 1).

## Results

In late 2019, the world faced a global crisis called COVID-19, which was a threatening epidemic. In addition to increasing the incidence and mortality of this epidemic, it caused significant other economic, social, political, and psychological problems. Social isolation by quarantining billions of people at home to disrupt the virus transmission chain has created many crises in various dimensions,(18) here are some of them:

Scope of knowledge and education. COVID-19 has disrupted students' lives in different ways and it has provided anxious times for students and parents.(19) According to UNESCO, more than 1.5 billion students in about 165 countries have been affected by the restrictions on schools and universities. As a result, schools, colleges and universities were forced to turn to online learning. This has caused students to continue their education at home.(20) Closing schools and accepting distance education may negatively affect students' learning through four main channels: spending less time learning, stress symptoms, changes in how students interact, and lack of motivation to learn. Most formal child learning takes place in schools, and closing schools and moving to a distance learning environment may cause children to spend less time learning.(21) Students who stay home because of COVID-19 are more likely to develop acute stress, maladaptation, and PTSD. The possibility of dropping out of school due to mental health problems also increases.(22) Attendance at schools increases the interaction between students and teachers and strengthens social skills and increases self-esteem and other skills necessary for the social environment. However, due to quarantine proceedings and the closure of schools, these social interactions have decreased.(21) Also, some teachers or students are not familiar enough with the world of the Internet and distance education, and this has disrupted the educational process. On the other hand, due to the lack of access to online education tools for all students such as phones or laptops or the lack of Internet access in less developed areas, problems in distance education have been created for them.(23)

Family scope. With the announcement of the epidemic of COVID-19 and the application of various restrictions in the community, some jobs were closed, which increased the financial and economic burden on the family and increased the couple's dissatisfaction, as well as disputes and even divorce. Also, due to the constraints and economic problems caused by COVID-19, weddings and bonds between people have decreased.(24, 25) With the closure of schools and the presence of students in homes and the closure of businesses due to social constraints, it has increased psychological problems in families, abuse and violence between family members.(26) This has a very important impact on people's lifestyle, the short-term consequences of which can be seen in health. These restrictions have led to decreased physical activity, weight gain, changes in diet, changes in smoking and alcohol consumption habits, changes in lifestyle, and ultimately quality of life and well-being.(27, 28) Also, different degrees of quarantine measures have reduced people's access to healthy food, inequality between communities, increased anxiety and stress, and impaired sleep quality. Some people also used drugs and alcohol to alleviate the fear and anxiety of COVID-19.(29)

Judicial scope. By imposing social distancing and various restrictions, individuals may resist these commands. The increasing burden of social distance in daily life has led to violence, bad temper, conflict, theft, murder, suicide, sabotage, and disregard for the law.(30) Social distance at the court level has also disrupted the proceedings and the presence of parties and witnesses in court.(31) The sale of illicit drugs, the distribution and consumption of drugs and alcohol, cybercrime, as well as the sale and purchase of firearms are among the crimes that are on the rise during COVID-19.(32) The outbreak of COVID-19 and the need for social isolation have also hampered the normal process of prisons. The temporary release of many prisoners in some countries has caused anxiety at the community level, and due to the lack of adequate space for solitary confinement, the possibility of increasing the incidence of COVID-19 among prisoners has become even more significant. Also, in some countries, the possibility of COVID-19 disease is higher among prisoners due to lack of adequate hygiene.(33)

Sexual scope. Anxiety about the state of the world, along with constant exposure to images of illness and death, has severely affected everyone's emotional stability. Daily turmoil, restriction of freedom and loss of sense of usefulness in society create a feeling of helplessness and overload in human beings. This has also affected the sexual sphere. Fear of these conditions has reduced physical contact in couples from simple kissing to full sexual intercourse and has weakened the bond between husband and wife.(34) Further depression and anxiety can reduce the level of sexual desire. With the closure of schools and the constant presence of children at home, sexual relations between individuals have also been overshadowed.(35) With the restrictions imposed, the tendency to have sex online has increased. Fear of infection has led single people to masturbate, have sex on the phone, and use of sexual means, which in turn has increased their desire for pornography and visits to pornographic sites and movies.(22^,^^36^)

Scope of transportation. Restrictions imposed by the government have halted public transport, restricted travel and activities abroad. The change of direction to personal transportation by individuals has intensified traffic on the roads and reduced the overall air quality in the urban environment. With the application of social distance, we are witnessing an increase in queues at bus and metro stations.(37^,^^38^) Air travel has also decreased significantly. COVID-19 has also led to the closure of many transportation agencies around the world due to a sharp drop in demand and a heavy economic burden on the people and the government. Violations and accidents on the road and in the city have also increased due to the use of private vehicles by the people.(16)

Cyberspace scope. This epidemic has played an important role in disseminating information in a news cycle.(39) The COVID-19 epidemic has not only posed significant challenges to the health care system worldwide, but has also played an important role in increasing rumors, deception and misinformation about the disease, its consequences, prevention and treatment. With so much news coming from different sources, there are many concerns about fake news. People are constantly following the news of COVID-19 and may experience high anxiety while doing things.(40) Comprehensive media exposure during the 24-hour news cycle can also lead the viewer to inaccurate and threatening information. These stress reactions may also have long-term consequences for physical and mental health.(41) It may even reduce the function of the immune system and upset the balance of their natural physiological mechanisms.(42)

## Discussion

The social effects of the changes resulting from the COVID-19 pandemic crisis are not yet well known. We know that due to the implementation of social distance policy, many of the usual activities of society in the economic, social and political spheres have been closed or suspended.(43) As a result, many people in areas such as business, family relationships, and education have experienced the changes and effects of this policy. Many sectors were forced to adjust their workforce and increase the number of unemployed to reduce costs.(44) The heavy economic burden and unemployment caused by the COVID-19 epidemic have caused anxiety thoughts, anxiety, and ultimately an increase in crimes such as theft, strife, domestic disputes, fraud, and etc. To prevent such crimes, the government can adopt policies that can include livelihood support, management and organization of Internet businesses, and low-interest lending.(45) In the policy of social distancing, the situation of certain social groups such as immigrants, refugees and addicts has been ignored and the problems of this group of people have intensified.(46) Civil society and voluntary groups of people are full of initiatives, innovations and resources that can be used in times of crisis. Internet infrastructures and modern communication tools have transcended the physical limitations of communication and enabled virtual social interactions that can be used to maintain connections.(47)

It should be noted that the lack of management and monitoring of the virtual world can impose irreparable damage on society in the long time.(3) Social distancing, despite its problems, has a significant impact on reduction of casualties from the disease and reducing its negative consequences, but ultimately it is the level of public trust in the government that ensures the success of policies and measures. It is necessary to provide more freedom of action for the media to provide accurate, transparent information in this regard. The development of information infrastructure, especially in the less developed regions of the country, must be seriously on the agenda. Necessary requirements for equipping students in need of teaching aids such as tablets.(11^,^^48^) The need for public transportation in any society is an indisputable need. Therefore, the government should take action to provide the desired service in accordance with social distance.(49) Increasing the number of public vehicles, regular disinfection and the use of social distance labels can revitalize this vital sector and meet the needs of the community and prevent pollution. Also, providing brochures and educational materials in public transportation can help reduce anxiety and increase public literacy.(16^,^^50^)

One must be careful about the social consequences of COVID-19 for certain groups. Ignoring this issue can cause COVID-19 to exacerbate the unfavorable situation of these groups. Groups such as addicts in crisis situations should be given more attention and clear and precise policies and programs should be developed to support this group. Immigrants and refugees also have their own circumstances, and their fate in the context of the COVID-19 pandemic crisis cannot be ignored. Doing all of the above means that a small part of the social issues created by the COVID-19 pandemic crisis require capacities of trust, cross-sectoral cooperation, coordination, transparency and joint action. Capacities that many of them may not have looked good under normal circumstances.(51^,^^52^)

Limitations of the study: In this study, due to the lack of proficiency of researchers in languages other than English and Persian, articles in other languages were excluded from the study, which can be a limitation of the study.

Conclusion: In this review study, the effect of coronavirus on some aspects of people's lives was briefly discussed. Unfortunately, in the current epidemic and control of the corona virus, while becoming a crisis of health in the world, it is considered as such that it still has various aspects. In recent months, the world has been going through one of the most severe crises in the field of health and without a doubt one of the most important consequences of its epidemic and social ills. In fact, anxiety factors related to the risk of contracting the disease, future employment status, and sources of income for individuals and families, as well as long-term quarantine, can be kept at home for a long time.

Implications of the results for nursing practice. Considering the psychological consequences of the COVID-19 outbreak, the design and planning of intervention and supportive strategies to reduce the negative effects are suggested. In fact, it is necessary to identify the factors that cause the danger to the psychological health of different people in the society in order to use appropriate treatment methods. 

**Table 1.** Details of articles used in research findings

Subject	Authors	Place and year	Type study	Samples	Results	Reference
Social isolation in COVID-19: The impact of loneliness	Banerjee D, Rai M	2020 India	Review	12 Articles	Social constraints in the COVID-19 pandemic force individuals to adapt to isolation and increase the prevalence of violence in the family, depression, anxiety, post-traumatic stress disorder.	(18)
Education and the COVID-19 pandemic	Daniel J	2020 Canada	Review	13 Articles	In order to increase the capacity of distance education, schools and colleges should use asynchronous learning and a variety of homework. Student assessment helps teachers provide flexible ways to compensate for students' learning deficiencies.	(53)
Global impact of COVID-19 on education systems	Osman ME	2020 Oman	Review	7 Articles	The normalization of current emergency e-learning does not necessarily mean extending the restrictions on face-to-face training, but rather pointing to strategies that control the prevalence of online acceptance at the time of COVID-19. It will ultimately change the learning landscape in schools and higher education institutions.	(20)
Impact of Pandemic COVID-19 on Education in India	Kumar Jena P	2020 India	Review	7 Articles	COVID-19 has greatly influenced India's education sector, and despite many challenges, various opportunities have evolved. They have explored the possibility of distance learning using digital technologies to cope with the COVID-19 crisis.	(54)
Student assessment of online tools to foster engagement during the COVID-19 quarantine	Lima KR, das Neves BHS, Ramires CC, Dos, et al	2020 Brazil	Descriptive Cross section	50 Students	To promote dynamic learning, various online tools are used for simultaneous and asynchronous training and activities. The course was evaluated well by students and they identified the use of Lt, Zoom, and YouTube platforms as the preferred online tools in learning physiology.	(23)
Risk and resilience in family well-being during the COVID-19 pandemic	Prime H, Wade M, Browne DT	2020 Canada	Review	110 Articles	Families face future threats due to the occurrence of COVID-19 in their relationships, laws, traditions and lifestyle, which can have major consequences for children's adjustment during this period. Some families will be more affected by previous circumstances (lower income families, mental health problems or special needs).	(24)
Decline in marriage associated with the COVID-19 pandemic in the United States	Wagner B, Choi K, Socius PC	2020 USA	Cross-sectional study	-	The number of marriages registered in 2020 is significantly lower than in the same period in 2019. It seems unlikely that the annual decline in marriages is due solely to the closure of government agencies that report marriage certificates.	(25)
Danger in danger: Interpersonal violence during COVID-19 quarantine	Mazza M, Marano G, Lai C, Janiri L, Sani G	2020 Italy	Review	17 Articles	During the outbreak of COVID-19, people faced an invisible and dark enemy and experienced disability. There is a need for programs aimed at preventing domestic violence and achieving an accurate assessment of several dimensions of abuse.	(26)
COVID-19 lockdown impact on lifestyle habits of Italian adults	Odone A, Lugo A, Amerio A, Borroni E, Bosetti C, , et al	2020 Italy	Cross sectional	6003 Participants	We have acquired a set of database analysis and are confident that we will achieve our research goals with the right budget and access to rich data resources.	(27)
Dietary and Lifestyle Changes During COVID-19 and the Subsequent Lockdowns among Polish Adults	Górnicka M, Drywié ME, Zielinska MA, Hamułka J	2020 Netherlands	Cross sectional	2381 Participants	Achieving a healthy pattern was negatively correlated with age but positively associated with pre-pandemic overweight. Living in macroeconomic areas reduced the achievement of a healthy model and increased the unhealthy model.	(28)
COVID-19 lockdown and lifestyles-A narrative review	Doraiswamy MBBS S, Cheema S, Al Mulla A, Mamtani R, Doraiswamy S	2020 Qatar	Review	649 Articles	Most articles have highlighted the negative impact of lockout measures on each of the lifestyle factors in many parts of the world. Such trends can positively affect the outcome of chronic lifestyle-related diseases, such as obesity and diabetes.	(29)
Impact of social distancing during COVID-19 pandemic on crime	Mohler G, Bertozzi AL, Carter J, Short MB, Sledge D, Tita GE, et al	2020 USA	Cross sectional	-	Measures of social distance may affect the extent and distribution of crime. recent study shows that social distance has a significant effect on several specific types of crime, but the overall effect is significantly less than expected.	(30)
The Changes In Criminal Trial Proceedings During COVID-19	Dewa Gede Giri Santosa	2020 Indonesia	Review	26 Articles	The results of this study address various issues that need to be addressed in order to change the criminal proceedings in COVID-19, and include not only the principle of expediency, but also the principles of justice and legal certainty.	(31)
COVID-19 Will Lead To Increased Crime Rates In India	Uppal P	2020 India	Review	11 Articles	The crime rate increases in such circumstances. To reduce it, the situation must be carefully monitored by law enforcement agencies. The past recession and changes in crime rates during and after the economic recovery were examined.	(55)
Corrections and Crime in Spain and Portugal during the COVID-19 Pandemic	Redondo S, Gonçalves RA, Nistal J, Soler C, Moreira JS, Andrade J, et al	2021 Spain	Cross sectional	225 Participants	The main mechanisms of COVID-19 transmission are the physical proximity between individuals and the shared use of contaminated equipment. Prisons must continue to maintain the most effective precautionary measures discussed in this article.	(33)
The impact of COVID-19 pandemic on pornography habits	Zattoni F, Gül M, Soligo M, Morlacco A, G, Collavino J, et al	2020 Italy	Review	43 Articles	The prevalence of COVID-19 has affected the use and consumption of pornography. After the national restriction, it was found that the search for online pornography and pornography related to the corona virus has increased.	(34)
COVID-19 and Sexuality: Reinventing Intimacy	Lopes GP, Vale FBC, Vieira I, da Silva Filho AL, Abuhid C, Geber S	2020 Brazil	Review	16 Articles	Social distance is still the best way to deal with COVID-19. It is recommended that couples living separately resume marital relationships, strengthening intimacy. A high-quality relationship is beneficial for physical, mental and sexual health. A chaotic and negative relationship causes stress and poor mental and sexual health.	(35)
Changes in Sexuality and Quality of Couple Relationship During the COVID-19	Panzeri M, Ferrucci R, Cozza A, Fontanesi L	2020 Italy	Cross sectional	124 Participants	The main reasons for the change in relationships in women seem to be concern, lack of privacy and stress. Even in participants with a high level of resilience, the negative aspects of locking up can affect the quality of sex.	(36)
Impact of the COVID-19 pandemic on the sexual behavior of the population.	Ibarra FP, Mehrad M, Di Mauro M, Peraza Godoy MF, et al	2020 Spain Italy Iran	Review	28 Articles	The impact of the Corona virus on the sexual life of individuals will be very important and in the coming months or years some relationships will change to some extent. Due to the many limitations of contact, the epidemic will negatively affect sexual behaviors.	(22)
Urban transport and COVID-19: challenges and prospects in low- and middle-income countries	Koehl A	2020 UK	Review	9 Articles	Cities have seen a steady decline in demand for transportation due to a combination of sustained economic crisis and changing work habits. This can lead to a change in behavior due to crowded spaces, especially public transportation.	(37)
Public transport during pandemic	Bandyopadhyay S	2020 India	Review	13 Articles	Previous activities and leisure have definitely challenged the way we learn. We have to educate ourselves in different ways and adopt our lifestyle accordingly. We must definitely go through the current difficult period and be ready to face any future challenges.	(38)
COVID-19 and Public Transportation: Current Assessment, Prospects, and Research Needs	Tirachini A, Cats O	2020 Chile	Review	69 Articles	Research needs related to the effects of the epidemic crisis on public transport. Some research needs (restoring the ability of public transportation systems to play their social role) require immediate attention.	(16)
The COVID-19 social media infodemic	Cinelli M, Quattrociocchi W, Galeazzi A, Valensise CM, Brugnoli E, et al	2020 Italy	Review	52 Articles	Understanding the social dynamics between content-consuming operating systems and social networks is an important research topic, as it may help design more efficient epidemic models for social behavior and design more effective communication strategies tailored to critical situations.	(56)
A New Application of Social Impact in Social Media for Overcoming Fake News in Health	Pulido CM, Ruiz-Eugenio L, Redondo G, Villarejo B	2020 Spain	Review	56 Articles	Messages that are based on evidence of respectful and transformative social impact, overcome misinformation about health. These results help advance knowledge in overcoming fake health-related news shared on social media.	(57)
Impact of rumors and misinformation on COVID-19 in Social Media	Tasnim S, Hossain M, Mazumder H	2020 USA	Review	10 Articles	The mass media, health care organizations, community-based organizations, and other key stakeholders need to build strategic partnerships and create shared platforms to deliver credible public health messages.	(58)
Facebook Pages Of Alternative News Media And The Corona Crisis-A Computational Content Analysis	Boberg S, Quandt T, Schatto-Eckrodt T, Frischlich L	2020 Germany	Review	70 Articles	Alternative news media remained faithful to the message patterns do not spread blatant lies, they mostly share overly critical, even anti-systemic messages, and oppose the views of the mainstream news media and the political apparatus.	(59)
The novel coronavirus outbreak: Amplification of public health consequences by media exposure	Garfin D, Silver R, Psychology EH-H	2020 USA	Review	22 Articles	Constant exposure of the media to the crisis of society can lead to increased anxiety and stress reactions that lead to effects on the flow of health, and health-protective behaviors can lead to over-reliance on health care facilities.	(41)
The impact of COVID-19 epidemic declaration on psychological consequences: a study on active Weibo users	Li S, Wang Y, Xue J, Zhao N	2020 Swiss	Review	36 Articles	People care more about health and family and less about leisure and friends. The use of social media data may provide a timely understanding of the impact of public health emergencies on people's mental health during an epidemic.	(42)
